# Editorial: Community-centric strategies for HIV and STI prevention in key populations

**DOI:** 10.3389/frph.2026.1798107

**Published:** 2026-03-16

**Authors:** Kathy Mngadi, Gail B. Broder

**Affiliations:** 1 Clinical Research Division, the Aurum Institute, Johannesburg, South Africa; 2 Vaccine and Infectious Disease Division, Fred Hutchinson Cancer Center, Seattle, WA, United States

**Keywords:** community-centric strategies, HIV prevention, implementation, PrEP uptake, protocol design, STI prevention, stigma

## Introduction

Key populations—women, adolescents, gender-diverse individuals, those who inject drugs, and historically underserved racial and ethnic groups—remain disproportionately vulnerable to HIV and STIs. Barriers include limited research designed for their needs, insufficiently trained staff, and the persistence of stigma and discrimination within healthcare settings (1, 2). Although biomedical innovation has accelerated, gaps persist in translating these advances into equitable access. Community involvement is often limited or introduced too late, resulting in prevention strategies that do not reflect the lived realities of those most affected (3).

This editorial summarizes the contributions of five manuscripts included in this Research Topic, each highlighting community-centered approaches that strengthen HIV and STI prevention across diverse global settings.

## Differentiated and demedicalized prevention models: lessons from Vietnam

Vietnam's national rollout of oral PrEP across 219 clinics in 29 provinces demonstrates how community engagement strengthens implementation. Collaboration with key population-led organizations guided the development of differentiated service models that improved confidentiality, reduced stigma, and addressed travel barriers. Month-3 continuation rates surpassed national averages as individuals selected service models aligned with their evolving preferences.

The Vietnamese experience underscores the importance of flexibility and user-driven choice, enabling the integration of new PrEP modalities as they emerge.

## Integrated, person-centered care for women who use substances

The HPTN 094 study focused on women who use drugs—a subgroup with overlapping needs related to addiction care, reproductive health, parenting responsibilities, unstable housing, and significant social stigma. A mobile health unit provided integrated services, including contraception, addiction treatment, HIV prevention, primary care, and peer navigation.

This dignified, non-judgmental model demonstrates that integrated care increases engagement and can improve recruitment and retention for future HIV-prevention research among highly marginalized populations.

## Policy and equity: a framework for historically black colleges and universities

Given the heightened vulnerability of young Black adults to HIV in the United States, the Office of HIV/AIDS Network Coordination (HANC) collaborated with Historically Black Colleges and Universities (HBCUs) to strengthen awareness of HIV prevention research. Activities included research-career exposure, improved health-science literacy, and structured conversations about stigma, structural racism, and historical mistrust.

A policy framework was developed to guide campus efforts to improve PrEP access, reduce prescriber bias, and integrate culturally sensitive HIV-prevention strategies. Increased awareness among students extended into their social networks on and beyond campus.

## Participatory practices in large-scale trials: insights from PrEPVacc

The PrEPVacc trial provides a strong example of effective Good Participatory Practice (GPP). Community advisory bodies were involved throughout the research lifecycle, and formative research guided communication. Animated materials improved understanding of informed consent and vaccine-induced seropositivity.

When one study arm was discontinued due to lack of efficacy, transparent communication preserved trust. As a result, the trial achieved its recruitment targets and maintained retention rates above 90%, demonstrating the direct impact of meaningful community partnerships.

## Gatekeepers, culture, and trust: lessons from South Africa

A qualitative study from South Africa explored how religious and traditional leaders shape perceptions of vaccines. Participants described these leaders as trusted sources of information who strongly influence decisions about COVID-19 vaccination. These findings are essential for HIV-vaccine preparedness—participants do not arrive as blank slates but rather bring socially mediated beliefs.

Engaging cultural and religious leaders as advocates could strengthen acceptance of future HIV and STI vaccines.

## Discussion

Across diverse settings, several consistent themes emerge:

1. Trust is central. Peer navigators, cultural leaders, community advisory boards, and transparent communication all strengthen trust, which directly influences the uptake of prevention services.
2. Flexibility and user-driven choice improve uptake. Differentiated service models, integration of care, and sensitivity to evolving preferences ensure that programs remain relevant.
3. Structural barriers must be addressed. Historical inequities, racism, stigma, lack of confidentiality, and logistical challenges continue to shape access. Addressing these barriers is essential for the equitable delivery of HIV prevention.
4. Community engagement enhances scientific quality. Trials designed with community input achieve better recruitment, retention, and relevance. Community-centered approaches help ensure that research findings translate effectively to real-world applications.

The manuscripts presented here collectively demonstrate that prevention succeeds when communities recognize themselves in the research design, the service delivery models, the messages, and the policies that guide uptake.

## Conclusion

This Research Topic highlights that sustainable progress in HIV and STI prevention depends on keeping communities at the center of research and implementation. By integrating lived experiences, prioritizing cultural sensitivity, and embedding trust-building practices into every stage of prevention work, these studies offer practical pathways toward more inclusive, effective, and equitable HIV-prevention strategies.
